# Molecular evolution of glutamine synthetase II: Phylogenetic evidence of a non-endosymbiotic gene transfer event early in plant evolution

**DOI:** 10.1186/1471-2148-10-198

**Published:** 2010-06-25

**Authors:** Sohini Ghoshroy, Manfred Binder, Aurélien Tartar, Deborah L Robertson

**Affiliations:** 1 Clark University, Biology Department, 950, Main Street, Worcester, MA 01610, USA; 2 Nova Southeastern University, 3301 College Avenue, Fort Lauderdale, FL 33314, USA

## Abstract

**Background:**

Glutamine synthetase (GS) is essential for ammonium assimilation and the biosynthesis of glutamine. The three GS gene families (GSI, GSII, and GSIII) are represented in both prokaryotic and eukaryotic organisms. In this study, we examined the evolutionary relationship of GSII from eubacterial and eukaryotic lineages and present robust phylogenetic evidence that GSII was transferred from γ-Proteobacteria (Eubacteria) to the Chloroplastida.

**Results:**

GSII sequences were isolated from four species of green algae (Trebouxiophyceae), and additional green algal (Chlorophyceae and Prasinophytae) and streptophyte (Charales, Desmidiales, Bryophyta, Marchantiophyta, Lycopodiophyta and Tracheophyta) sequences were obtained from public databases. In Bayesian and maximum likelihood analyses, eubacterial (GSII_B_) and eukaryotic (GSII_E_) GSII sequences formed distinct clades. Both GSII_B _and GSII_E _were found in chlorophytes and early-diverging streptophytes. The GSII_B _enzymes from these groups formed a well-supported sister clade with the γ-Proteobacteria, providing evidence that GSII_B _in the Chloroplastida arose by horizontal gene transfer (HGT). Bayesian relaxed molecular clock analyses suggest that GSII_B _and GSII_E _coexisted for an extended period of time but it is unclear whether the proposed HGT happened prior to or after the divergence of the primary endosymbiotic lineages (the Archaeplastida). However, GSII_B _genes have not been identified in glaucophytes or red algae, favoring the hypothesis that GSII_B _was gained after the divergence of the primary endosymbiotic lineages. Duplicate copies of the GSII_B _gene were present in *Chlamydomonas reinhardtii*, *Volvox carteri *f. *nagariensis*, and *Physcomitrella patens*. Both GSII_B _proteins in *C. reinhardtii *and *V. carteri *f. *nagariensis *had N-terminal transit sequences, indicating they are targeted to the chloroplast or mitochondrion. In contrast, GSII_B _proteins of *P. patens *lacked transit sequences, suggesting a cytosolic function. GSII_B _sequences were absent in vascular plants where the duplication of GSII_E _replaced the function of GSII_B_.

**Conclusions:**

Phylogenetic evidence suggests GSII_B _in Chloroplastida evolved by HGT, possibly after the divergence of the primary endosymbiotic lineages. Thus while multiple GS isoenzymes are common among members of the Chloroplastida, the isoenzymes may have evolved via different evolutionary processes. The acquisition of essential enzymes by HGT may provide rapid changes in biochemical capacity and therefore be favored by natural selection.

## Background

Glutamine synthetase (GS: E.C. 6.3.1.2) catalyzes the ATP-dependent formation of Gln from Glu and NH_4 _^+ ^and is considered one of the oldest functioning enzymes [1,2]. The GS gene superfamily includes three distinct classes, GSI, GSII and GSIII, each differing in molecular size and number of subunits in the holoenzyme [3,4]. The distribution of the three classes is variable within the three domains of life and instances of multiple GS isoenzymes from different families functioning in the same organism are not uncommon in both eubacteria and eukaryotes [3,5-8]. These observations suggest the gene families arose early and prior to the divergence of the prokaryotes and eukaryotes [9-11].

The evolutionary history of the GS superfamily is complicated and gene transfer events are one among many forces that have shaped the history of these genes. Among prokaryotes, there is phylogenetic evidence of horizontal gene transfer (HGT) of GSI genes between members of the Archaea and Eubacteria [9]. Evidence for secondary endosymbiotic gene transfer of GSII from the red algal endosymbiont to the nucleus of the heterokont host has also been presented [11]. Members of the GSII gene family are found in both eubacterial and eukaryotic lineages. The identification of GSII genes in the plant symbiont *Bradyrhizobium japonicum *lead Carlson and Chelm [12] to hypothesize that the gene evolved via HGT from vascular plants to bacteria. However, this hypothesis was not supported by subsequent phylogenetic analyses [11,13], which established distinct eukaryotic (GSII_E_) and eubacterial (GSII_B_) clades.

The supergroup Archaeplastida [14], consisting of Glaucophyta, Rhodophyceae and Chloroplastida, harbors members of GSII gene family that are well characterized in vascular plants but not in other lineages within the supergroup. In general, vascular plants express multiple GS isoenzymes that are localized to either cytosol or chloroplast. The isoenzymes are nuclear encoded, and in most angiosperms a single nuclear gene encodes the chloroplast isoenzyme, while a small nuclear gene family encodes multiple cytosolic isoenzymes that are expressed in tissue-specific and developmentally-regulated patterns [15-18]. Previous phylogenetic analyses of chloroplast and cytosolic isoenzymes support the hypothesis that the isoenzymes in angiosperms evolved via a gene duplication event that preceded the divergence of monocots and dicots [19,20].

Biochemical studies of green algae provided the first evidence that, as observed in vascular plants, multiple GSII isoenzymes are expressed and localized to the cytosol and chloroplasts within these organisms [21,22]. Phylogenetic analyses incorporating the two GSII isoforms characterized in *Chlamydomonas reinhardtii *[23] uncovered an unusual disparity between the two enzymes [20]. The cytosolic GSII sequence clustered with the vascular plants while the plastid sequence branched more basally and appeared to associate with the eubacterial sequences.

Here we examined the evolutionary relationship of the GSII gene family and use increased taxonomic sampling in Chloroplastida to determine if the basally branching, eubacterial-like GSII_B _was broadly distributed. GSII sequences were obtained from four members of the Trebouxiophyceae (Chlorophyta) by PCR amplification using degenerate and gene specific primers. Additional GSII sequences for members of the green algae (Chlorophyta and Prasinophytae) and streptophytes (*Mesostigma*, Charales, Desmidiales, Bryophyta, Marchantiophyta, Lycopodiophyta and Tracheophyta) were obtained from publicly available databases, including genome and EST projects. We also increased taxonomic sampling within Eubacteria (to date, GSII genes have not been reported from Archaea). GSII_E _and GSII_B _sequences were identified in members of the green algae and early-diverging streptophytes. Phylogenetic analyses provide support for the hypothesis that GSII_B _was gained in the Chloroplastida from the Eubacteria via a HGT event after the divergence of primary photosynthetic groups.

## Results and Discussion

### Amplification of GSII genes

Complete GSII mRNA sequences were obtained from *Pseudochlorella *sp. CCAP211/1A, *Chlorella luteoviridis*, *Auxenochlorella protothecoides*, and *Prototheca zopfii*. A GSII sequence was also obtained for *Pseudochlorella *sp. CCAP211/1A that included 912 bp of the ORF and all of the 3'UTR. GenBank accession numbers and characteristics of the transcripts obtained in this study are summarized in Table 1.

**Table 1:** Summary of the GSII sequences characterized in the present study

Taxa	Sequences Obtained				% GC content		
	**Accession Number**	**Length (bp)**	**ORF (bp)**	**Amino Acids**	**ORF**	**5' UTR**	**3' UTR**

**GSII_E _Sequences**							
*Pseudochlorella *sp. CCAP211/1A (2)	GQ465769	1486	1137	378	63.32	53.19	55.12
*Chlorella luteoviridis *UTEX 28	GQ465770	1675	1146	381	56.20	46.27	46.17
*Auxenochlorella protothecoides*	GQ465771	1621	1161	386	67.96	58.18	67.28
*Prototheca zopfii *ATCC16527	GQ465772	1632	1158	385	69.26	71.54	72.42

**GSII_B _Sequences**							
*Pseudochlorella sp*. CCAP211/1A (1)	GQ491030	1266	912	303	56.47	n.d.	47.93

Four complete sequences of GSIIE and a portion of one GSIIB were obtained from cDNA for the species listed above. NCBI (GenBank) accession numbers are given. Characteristics of the sequences in terms of nucleotide length (Length), size of open reading frame (ORF), and the length of the predicted amino acid sequences (Amino Acids) are presented. The % GC content of open reading frame (ORF) and the 5'and 3' untranslated regions (UTR) of each transcript are presented.

### Eukaryotic GSII phylogeny

**Figure 1:**
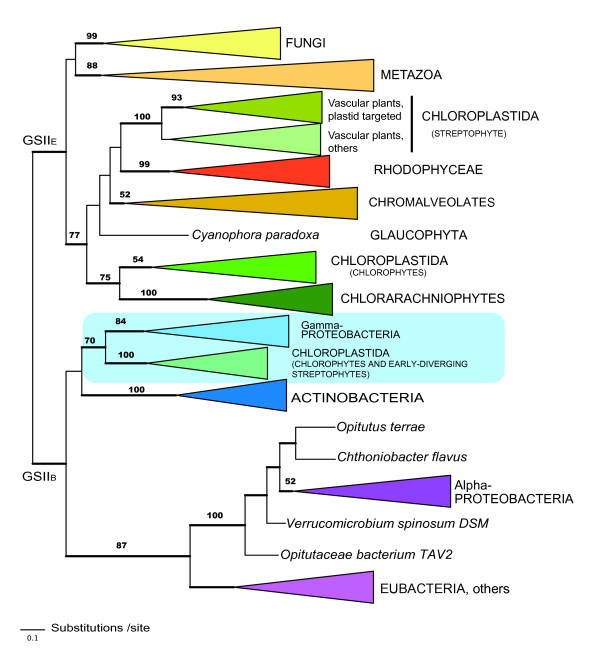
**Evolutionary relationships among GSII enzymes from prokaryotes and eukaryotes**. The phylogenetic analyses were based on 333 amino acid characters from 196 taxa. The 50% majority-rule consensus tree from the Bayesian analyses [48,49] is shown as inferred from 20,002 trees as described in the Methods. Nodes with BBP support > 0.95 are represented by thick lines. RAxML [50,51] bootstrap values are indicated for nodes recovered in both analyses. RAxML values are not indicated for terminal bifurcations. Eubacterial GSII_B _were used as the outgroup and considered monophyletic. The area of the triangles representing collapsed clades is not proportional to the number of taxa within the clade.

**Figure 2:**
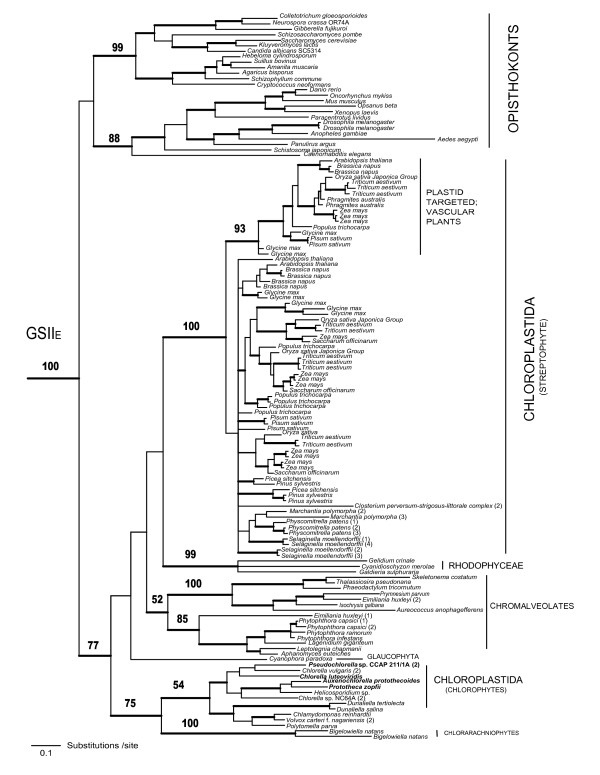
**Evolutionary relationship of GSII_E _genes from eukaryotes, terminal taxa expanded**. Phylogenetic analyses are as described in figure 1. Nodes with BBP support > 0.95 are represented by thick lines. RAxML bootstrap values are indicated for major nodes. RAxML values are not indicated for terminal bifurcations. Sequences characterized in the present study are shown in bold.

Phylogenetic analyses of GSII amino acid sequences resulted in a well-resolved tree. Assuming the root of the tree lies outside the major eukaryotic clade, there was a clear separation of the eukaryotic (GSII_E_) and eubacterial (GSII_B_) enzymes (Figure 1). Within the eukaryote clade, the opisthokonts (fungi+animals) and photosynthetic eukaryotes formed separate groups (Figures 1 and 2). The GSII_E _proteins from streptophytes, chlorophytes, rhodophytes, and chromalveolates formed distinct clades, each with strong to moderate support. The position of the heterokont sequences within this clade is consistent with previous analyses that provide evidence that GSII_E _in heterokonts arose via endosymbiotic gene transfer [11]. Sequences from Chlorophyta (green algae, including representatives of the Chlorophyceae and Trebouxiophyceae) diverged from a basal node within the photosynthetic clade and the Chloroplastida (eukaryotes with chlorophylls *a *and *b*) were not monophyletic. However, the deeper nodes within the photosynthetic eukaryotic clade were not well supported and thus the branching pattern within the clade is unresolved (Figure 2). The streptophyte GSII_E _sequences formed two major groupings; one group contained protein sequences targeted to the chloroplast of angiosperms and the other contained protein sequences of non-vascular and vascular plants that are targeted to the cytosol. Multiple GSII_E _genes were also observed in the gymnosperms (*Pinus *spp.) but to date, these appear to function in the cytosol and evidence of plastid targeted isoenzymes is lacking [24].

### Evidence for the HGT of GSII_B_

**Figure 3:**
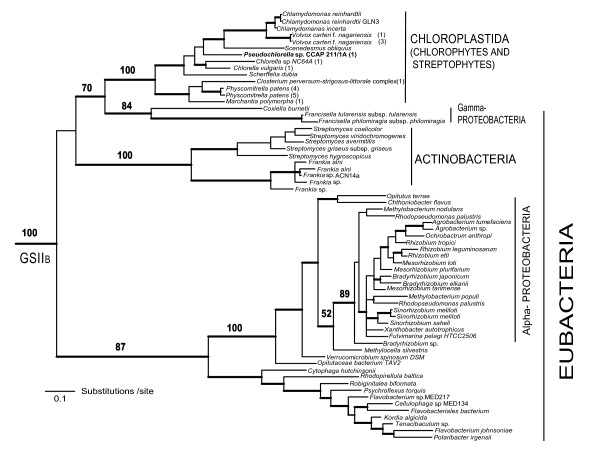
**Evolutionary relationship of GSII_B _genes from prokaryotes and eukaryotes, terminal taxa expanded**. The inclusion of GSII_B _genes from eukaryotes (early-diverging Chloroplastida) within prokaryotic GSII_B _clade is evidence of HGT from prokaryotes to eukaryotes within this group. Nodes with BBP support > 0.95 are represented by thick lines. RAxML bootstrap values are indicated for major nodes. RAxML values are not indicated for terminal bifurcations. The sequence characterized in the present study is shown in bold.

The GSII_B _clade comprised sequences from eubacteria and some members of the Chloroplastida (green algae, liverworts, and mosses; Figures 1 and 3). The Chloroplastida sequences formed a single clade nested within the eubacterial sequences and branching within the clade was similar to predicted organismal phylogenies [25].

GSII_B _sequences are not broadly represented among eubacteria but were identified in members of the Bacteriodetes/Flavobacteria/Cytophaga, Planctomycetes, Verrucomicrobia, Actinobacteria, and the α- and γ-Proteobacteria (Figure 3; Additional files 1 and 2). The Chloroplastida GSII_B _was sister to γ-Proteobacteria with strong (Bayesian posterior probability = 1.0) to moderate support (likelihood bootstrap support = 70%). The γ-Proteobacteria + Chloroplastida GSII_B _clade was sister to the Actinobacteria, but this association was not strongly supported. The α-Proteobacteria GSII_B _sequences were not closely related to the γ-Proteobacteria + Chloroplastida GSII_B _clade, which makes the possibility of GSII_B _gain via mitochondrial endosymbiosis unlikely. The α-Proteobacteria GSII_B _were nested within the Verrucomicrobia and thus, we cannot exclude the possibility of an HGT event within the α-Proteobacteria lineage that obscures the mitochondrial origin of the GSII_B _gene in the Chloroplastida. However, the lack of detection of GSII_B _in genomes of other eukaryotic lineages reduces the likelihood of a mitochondrial origin. In addition, EST and genome analyses of other photosynthetic eukaryotes (Glaucophyta, Rhodophyceae and Chromalveolates) and extant cyanobacteria [26,27], have not uncovered GSII_B _sequences, reducing the possibility that GSII_B _was acquired via plastid endosymbiosis. Thus, we propose that GSII_B _in the Chloroplastida arose via a HGT from γ-Proteobacteria early in plant evolution.

GSII_B _sequences are not broadly distributed among eubacterial lineages and to date, within γ-Proteobacteria, only the genera represented in our analyses have annotated GSII_B _sequences deposited in GenBank. Assuming the GS superfamily evolved prior to the divergence of the three domains of life [9-11], the distribution of GSII_B _sequences suggests the gene has been lost in several lineages of Eubacteria and the Archaea. The analysis of GSII_B _may become more robust as additional eubacterial GSII_B _become available through genome sequencing projects. However, gene loss may make the identification of the true donor of GSII_B _to the Chloroplastida difficult.

An alternative explanation for the limited distribution of GSII_B _among the eubacteria is that the gene was transferred to the eubacteria from an eukaryotic donor. The possibility of an HGT from Chloroplastida to the γ-Proteobacteria is not supported by our phylogenetic analyses as it implies that the eubacterial sequences would nest within the GSII_E _clade; which has not been observed in our phylogenetic analyses. Eukaryote to eubacterial HGT might be supported if GSII_B _were found in diverse lineages of eukaryotes. Further investigation of GSII diversity in the eukaryotic lineages not represented in our study (e.g., Rhizaria, Excavata and Amoebozoa) will contribute to our understanding of the distribution and evolution of GSII_B_. Given the data at hand, however, the hypothesis that GSII_B _arose in the Chloroplastida via HGT remains the most parsimonious.

### Estimating the timing of the HGT

**Figure 4:**
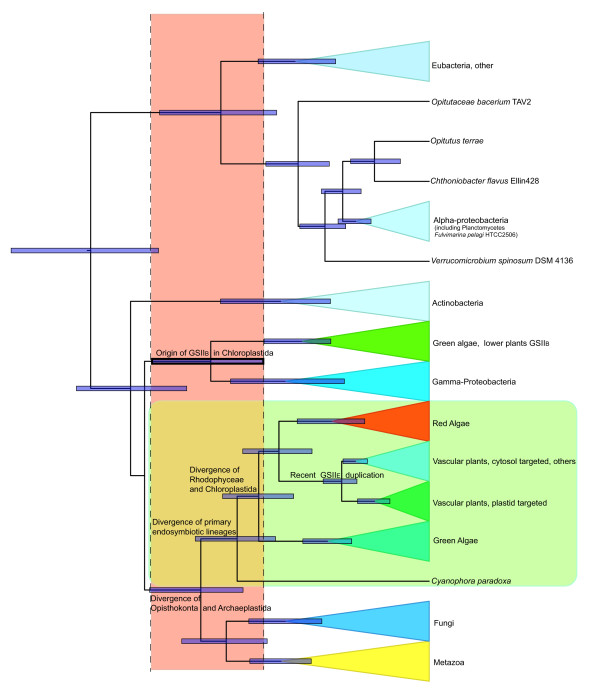
**Maximum clade probability chronogram from the BEAST analysis of the GSII_B _and GSII_E _amino acid sequence alignment**. All lineages were allowed to evolve according to a relaxed molecular clock and WAG + Inv + Gamma model. Bars on nodes indicate the width of the 95% highest posterior density for each divergence time.

To estimate the relative and absolute timing of the HGT of GSII_B_, we used Bayesian relaxed molecular clock analyses [28]. Both the uncalibrated (Figure 4) and calibrated analyses (Additional file 3) show an overlap of the 95% highest density posterior node ranges of the origin of GSII_B _in the early-diverging Chloroplastida coinciding with the GSII_E _divergence in the opisthokonts and in the primary photosynthetic eukaryotes (Archaeplastida). Our analyses indicate that GSII_B _and GSII_E _may have coexisted for an extended period of time and under this scenario, the putative timing of the HGT event from eubacteria to eukaryotes could be placed either prior to or after the divergence of the primary photosynthetic lineages. At present, there is no evidence of GSII_B _in genomes of red algae (*Cyanidioschyzon merolae *[29] and *Galdieria sulphuraria *[30,31]), or the glaucophyte *Cyanophora paradoxa*. We acknowledge that taxon sampling is not extensive within these two lineages and hence cannot exclude the possibility of the existence GSII_B _in these groups. However, given these limited data it is most parsimonious to assume that GSII_B _was acquired only by the Chloroplastida, early after the divergence from the Glaucophyta and Rhodophyceae (red algae).

The distribution of GSII_B _within Chloroplastida covers the major lineages of Chlorophyta (Chlorophyceae, Trebouxiophyceae, and Prasinophyceae; Additional file 1). In addition, a partial GSII_B _sequence was identified in a member of the Ulvophyceae (*Acetabularia acetabulum*; (Additional files 2 and 4). Within Streptophyta, GSII_B _genes are present in Mesostigmatophyceae (*Mesostigma viride*; Additional files 2 and 4), Zygnemophyceae (Desmidiales; *Closterium peracerosum-strigosum-littorale *complex), Marchantiophyta (liverworts; *Marchantia polymorpha*) and Bryophyta (mosses; *Physcomitrella patens*). GSII_B _is absent from the single Lycopodiophyta genome (*Selaginella moellendorffii*) and from all seed plants. Hence, we propose that GSII_B _was lost in the plant lineage after the colonization of land by early plants, marked by the divergence of bryophytes and lycopodiophytes, which is one of the oldest vascular plant lineages [32].

### Functional localization and GSII_B _gene duplication

The Chloroplastida lineages that contain the GSII_B _gene also have a GSII_E _counterpart, which attaches to a basal node within the photosynthetic eukaryotes (Figure 1). Both the GSII_B _and GSII_E _genes are nuclear encoded and thus we identified the cellular location of each of the gene products based on the presence (organelle-localized) or absence (cytosol-localized) of N-terminal transit peptides using TargetP ver. 1.1 ([33], see Additional file 5). None of the early-diverging Chloroplastida GSII_E _enzymes contained transit peptides. In contrast, chloroplast transit sequences were identified in the GSII_B _protein sequences from *Chlorella *sp. NC64A, *C. vulgaris *and the streptophyte, *Closterium peracerosum-strigosum-littorale *(Zygnemophyceae) but not in the moss (*P. patens*) or liverwort (*M. polymorpha*). Mitochondrial-targeting transit peptides were predicted in GSII_B _sequences from *C. reinhardtii*, *Volvox carteri *f. *nagariensis *and *Scenedesmus obliquus *(see Additional file 5). Previous work indicated that chloroplast transit sequences from *C. reinhardtii *shared features with both mitochondrial and higher plant chloroplast pre-sequences [34] and thus the prediction of a mitochondrial location of GSII_B _may not reflect its true functional localization. Alternatively, GSII_B _may be targeted to both the mitochondria and chloroplast, similar to what is observed for GSII_E _in leaves of some vascular plants [35,36]. While experimental evidence is required to confirm the cellular localization of the GSII_B_, it appears that the GSII_B _enzymes function in either the chloroplast or mitochondrion in the chlorophytes and early-diverging streptophytes (*Closterium *sp.) and that GSII_E _functions in the cytosol.

The GSII_B _gene is duplicated in *C. reinhardtii, V. carteri *f *nagariensis *and *P. patens*. The duplicated copies of GSII_B _in *C. reinhardtii *and *V. carteri *f *nagariensis *were nearly identical (90% and 95% identical, respectively) and present in the genome in a head-to-head orientation. Similarly, the GSII_B _genes in *P. patens *were 98% identical but do not appear to be in close genomic proximity. Within our phylogenetic analyses (Figure 3), the duplicated GSII_B _of *C. reinhardtii*, *V. carteri *f. *nagariensis *and *P. patens *each formed separate clades, suggesting the genes evolved by independent duplication events. Alternatively, the GSII_B _genes in *C. reinhardtii *and *V. carteri *may have evolved via an early duplication within the Chlamydomonadales with subsequent gene conversion following the divergence of these lineages. The GSII_B _are differentially expressed in *C. reinhardtii *suggesting the need for maintenance of both the copies in the organism [37].

### GSII_B _loss and replacement of function

In contrast to the expression of GSII_E _and GSII_B _genes in the early-diverging Chloroplastida, the chloroplast- and cytosolic-localized GSII enzymes in angiosperms are both members of the GSII_E _family and form two distinct clades in our phylogenetic analyses (Figures 1 and 2). As predicted in earlier studies [19], the genes encoding these enzymes arose via a recent gene duplication event with further expansion in the number of genes encoding cytosolic isoenzymes in several plant lineages (Figure 2, [38,39]). Since GSII_B _is absent from vascular plants, it appears that the chloroplast function of GSII_B _has been replaced by a gene duplication event in higher plants allowing for subsequent loss of the gene from this lineage. There is also an expansion of the GSII_E _gene family in gymnosperms (Figure 2), but the enzymes are all localized to the cytosol and the plastid targeted isoform appears to have been lost from this group. The expansion of the GSII_E _gene family coincides with the development of vascularization of land plants and maybe correlated with the partitioning of nitrogen assimilation between below and above ground tissue (see Additional file 3).

## Conclusions

We have provided evidence of an ancient HGT event involving the gene for an essential enzyme, GSII. GSII has been well characterized at the molecular level in angiosperms but has been largely overlooked in the early-diverging plant lineages, which were addressed in the present study. Although recent comparative genomic analyses failed to identify bacterial genes in *Chlamydomonas reinhardtii *[40], our discovery of a eubacterial-like GSII in the chlorophytes and early-diverging streptophytes suggests that further exploration within these lineages is merited. The branching pattern within the monophyletic assemblage of the chlorophytes and early-diverging streptophytes is similar to other molecular and organismal phylogenies, suggesting the occurrence of a single HGT event. As a result, GSII_B _may be useful in resolving taxonomic associations within and among green algal and early-diverging streptophyte lineages.

Several genes of bacterial origin have been identified in *Dictyostelium discoideum *and are thought to be advantageous to organisms living in soil [40]. More recently, Richards et al. [41] identified five genes in plants that appear to be of fungal origin and argue that two may have been advantageous for organisms colonizing a terrestrial environment. We propose that the acquisition of enzymes by HGT results in a more rapid change in enzymatic capacity or kinetic diversity than evolution of isoenzymes by gene duplication and subsequent specialization. Biochemical studies have suggested that GSII_B _has a lower affinity for NH_4 _^+ ^and Glu than GSII_E _[42], characteristics that would be advantageous for enzymes assimilating higher concentrations of NH_4 _^+ ^from environmental sources, NO_3 _^- ^assimilation, or increased rates of photorespiration. Increased taxon sampling and an enlarged fossil age constraint dataset will allow for a more detailed examination of the timing of GSII gains and losses over geological history and coupled with major transitions in plant evolution.

## Methods

### Algal cultures and sequencing

Four members in the class Trebouxiophyceae were selected for GSII gene amplification. Cultures of *Pseudochlorella *sp. CCAP211/1A, *Chlorella luteoviridis*, and *Auxenochlorella protothecoides *were a gift from Dr. Peggy Winter (University of West Florida), and *Prototheca zopfii *was a gift from Dr. Drion Boucias (University of Florida). Cultures were grown axenically in ATCC medium 847, (*Pseudochlorella *sp. CCAP211/1A, *C. luteoviridis *and *A. protothecoides*) and in ATCC medium 28: Emmons' modification of Sabouraud's agar (*P. zopfii*) at 17°C and 12:12 h light: dark cycle. Cells were collected by centrifugation (approximately 50 mL of culture), flash frozen in liquid nitrogen, ground in a mortar and pestle and subjected to DNA and RNA extraction. DNA was extracted using a hexadecyltrimethylammonium bromide extraction protocol [43]. RNA was extracted using an RNeasy^® ^Mini Kit (Qiagen Inc., Valencia, CA) with modifications outlined in Brown et al. [44] for extraction with glass beads using bead beating. Extracted nucleic acids were quantified spectrophotometrically for downstream applications using a MWG BIOTECH Lambda Scan 200×, 96-well Microplate Reader with KCJunior Software (MWG BIOTECH, High Point, NC). cDNA was synthesized using an Omniscript RT kit (Qiagen Inc., Valencia, CA). Total RNA (1.5 μg) was used as a template and the oligo-d (T) primer GCGGCCGCTCTAGACTAG(T)_18 _as the first strand primer. Primers were designed to target specifically GSII_E _and GSII_B _sequences. GSII_E _primers were based on existing sequences from vascular plants, chlorophytes and rhodophytes. GSII_B _primers were based on existing sequences from *Chlamydomonas reinhardtii *and *Physcomitrella patens*. PCR was performed in a final volume of 25 μL with Taq PCR core kit (Qiagen) with Q solution to overcome problems associated with high GC content. Primer sequences are listed in Table 2. Thermal conditions for GSII_E_: 30 cycles of 95°C for 30s, 50°C for 30s, 72°C for 1 min, performed for 30 cycles. Thermal conditions for GSII_B_: Initial denaturation of 94°C for 2 min, followed by 35 cycles of 94°C for 1 min, 51°C for 1 min, 72°C for 1 min and extension at 72°C for 5 min.

**Table 2:** Primers used for amplification of GSII genes from green algae

Gene	Primer name	Direction	Sequence
	MossGS2-1F	Forward	5'-TGGGTTGATGGTMANGARGG-3'
GSII_B_	MossGS2-2R	Reverse	5'-ATNCCGAAMTCTTCNCC-3'
	Green UNI 1-F	Forward	5'-CCIRAITGGWSITTYGAYGG-3'
	cpGSII(QGPFY)-R	Reverse	5'-CCRCARTARAAIGGICCYTGIGG-3'

	GALG GS F	Forward	5' - TGC CCA TCC CCA CCA ACA C - 3'
GSII_E_	GALG GS R	Reverse	5' - TCT CGT GCT TGC CCG TCA GG - 3'
	GS2ChloroF	Forward	5' - CGG CWT CGA GCA GGA GTA CAC - 3'
	GS2ChloroR	Reverse	5' - CCG AYC TGG WAC TCC CAC TGG - 3'

Sequences of degenerate primers are presented using IUBMB single letter codes. I represents inosine.

Nested PCR amplification was used to obtain GSII_B _sequences with first round done with cDNA and primer concentrations of 0.4 μM (MossGS2-1F [forward] and MossGS2-2R [reverse]). The amplicon was used for a second round of amplification with primers Green UNI 1-F (forward) and cpGSII(QGPFY)-R (reverse) and yielded a DNA fragment of 330 bp. Amplified sequences were cleaned and sequenced either commercially (MWG Biotech, Charlotte, NC and Macrogen, Seoul, South Korea) or at Clark University using an automated DNA sequencer (ABI 3130), with ABI Prism Terminator Big Dye ver 3.1 (Applied Biosystems, Carlsbad, CA). Some PCR products were cloned into TOPO vectors following the manufacturer's protocol (TOPO TA Cloning Kit for Sequencing, Invitrogen, Carlsbad, CA) prior to sequencing. Rapid Amplification of cDNA Ends (RACE) methods were used to obtain the entire open reading frame for GSII_E _sequences from *Pseudochlorella *sp. CCAP211/1A, C. *luteoviridis*, *A. protothecoides *and *P. zopfii *and partial GSII_B _sequence from *Pseudochlorella *sp. CCAP211/1A. 3' RACE reactions used a combination of gene specific primers and a portion of the oligo-d (T) primer (GCGGCCGCTCTAGACTAGT) used for cDNA synthesis. 5' RACE reactions were performed using 5' RACE System version 2.0 from Invitrogen (Invitrogen) and SMART™ RACE cDNA Amplification Kit (Clontech Laboratories Inc., Mountain View, CA) following manufacturers' recommendations. Contigs were assembled using CodonCode Aligner (CodonCode Corporation, Deadham, MA). All sequences were translated into amino acids *in silico*.

### Phylogenetic analyses

GSII sequences were retrieved from public databases as well as genome and EST projects using the GSII sequence from the diatom *Skeletonema costatum *(AAC77446) as query, or glutamine synthetase as a keyword. Subsequent queries with eubacterial GSII sequences did not retrieve any additional sequences. Complete information on taxa, database sources and accession numbers is provided in Additional file 1. The initial alignment of amino acid sequences was done with the web based program CLUSTAL W, using default parameters [45], followed by manual adjustment using BioEdit Sequence Alignment Editor [46] and MacClade 4.08 [47]. The N- and C terminal ends of the proteins along with highly variable regions within the alignments were excluded in the phylogenetic analyses.

The final GSII alignment consisted of 196 taxa and 333 characters for Bayesian analysis. Trees were inferred by calculating Bayesian posterior probabilities using MrBayes 3.1.2 [48,49]. Two parallel runs, each with four chains (three heated and one cold) were run for 10^6 ^generations. The evolutionary models implemented in MrBayes3.1.2 were explored using the mixed amino acid model. Rate variation across sites was approximated using a gamma distribution with proportion of invariable sites estimated from the data. Trees were sampled every 100 generations. Likelihood tree scores of two independent runs were plotted to estimate the point of convergence to a stable likelihood, and to determine the trees to be excluded via "burnin." Bayesian posterior probabilities of the branches were calculated from trees from both the runs, totaling 20,002 trees. Trees remaining (10,000) after a burnin of 5001 for each run were used to compute a 50% majority-rule consensus.

Maximum likelihood (ML) based inference of the phylogenetic trees was done using the software RAxML 7.0.4 [50,51]. The analysis used a random starting tree and the rapid hill-climbing algorithm (i.e., option -f d in RAxML) and the WAG model of amino acid substitution were used. A random seed number was used to turn on rapid bootstrapping (-x) and 1000 bootstrap trees were generated by invoking -# 1000 and - x options in RAxML. A majority rule consensus tree was created in PAUP* 4.0 b [52]. The phylogenetic trees in figures 1, 2 &3 are the 50% majority rule consensus trees from the Bayesian analyses on which the RAxML bootstrap values have been indicated. The eubacterial GSII_B _sequences were used as the monophyletic outgroup in the graphical representation of the phylogenies.

### Prediction of functional localization of GSII_B _and GSII_E _protein sequences in early-diverging Chloroplastida

We used the web-based programs TargetP 1.1[33] and ChloroP 1.1 [53] to identify N-terminal transit peptides in GSII_B _and GSII_E _proteins (see Additional file 5).

### Estimation of divergence times

We estimated the divergence times using Bayesian approach implemented in BEAST 1.4.8 [28]. We did an un-calibrated and calibrated run. A relaxed molecular clock model of uncorrelated log normal distribution was used. For the un-calibrated analysis, a starting tree generated by RAxML 7.0.4 [50] was used as the input tree with the GS amino acid sequence alignment. For the calibrated analysis we set uniform priors on tmrca parameter. Fossil dates were used as minimum dates and were, as follows, Ascomycota, 400 MYA [54], Bilateria, 550 MYA [55] and streptophytes 475 MYA [56]. Secondary age constraints based on published estimates of divergence times were not used. We used the following models, WAG + Inv + Gamma with priors, birth death speciation on the tree. Markov Chain Monte Carlo was set to default 10 million with sampling at every 1000 generation, resulting in 10,000 trees. Convergence was assessed in Tracer v 1.4 [57] and the first three million samples were excluded as burnin. A maximum clade credibility tree was generated by analyzing the BEAST tree file in TreeAnnotator 1.4.6 [58]. This program determined the 95% highest posterior densities and estimated the node heights as mean heights.

## Abbreviations

GS: Glutamine synthetase; HGT: Horizontal gene transfer; BPP: Bayesian posterior probability;

## Authors' contributions

SG participated in the cloning and sequencing of GSII genes, assembled the data matrix, conducted all phylogenetic analyses, and drafted the manuscript. MB participated in the execution and interpretation of the relaxed molecular clock analyses and assisted in the writing of the manuscript. AT was responsible for the cloning and sequencing of GSII genes and assisted in writing the manuscript. DLR conceived of the study, coordinated the research, and helped draft the manuscript. All authors read and approved the final manuscript.

## Supplementary Material

Additional file 1 **GSII protein sequences used in the present study**. GenBank accession numbers and JGI DOE scaffold and protein ID information for the GSII proteins are provided. Click here for file

Additional file 2 **Identification of GSII_B _sequences from *Acetabularia acetabulum *and *Mesostigma viride***. The data matrix used in this analyses is described in the Methods with sequences from the following taxa included in the alignment: *Acetabularia acetabulum *(Chlorophyta, Ulvophyceae), *Mesostigma viride *(Streptophyta, Mesostigmatophyceae), which are shown in bold. *Pseudochlorella *sp. CCAP211/1A (1) GSII_B _amplified in the present study is also shown in bold. Tree used for this illustration is a representative derived from parsimony heuristic search analysis. RAxML bootstrap values are shown for the major clades of GSII genes, which were derived from 1000 bootstrap replicates with the parameters described in the Methods. Click here for file

Additional file 3 **Maximum clade probability tree displayed as a chronogram from the BEAST analysis of the GS amino acid sequence alignment**. All lineages evolved according to a relaxed molecular clock and WAG + Inv. + Gamma model. Node bars indicate the width of the 95% highest posterior density with minimum and maximum values in parentheses. Bold numbers near the nodes indicate node ages. Major lineages are depicted as collapsed triangles. Nodes for which fossil dates were used are marked as A = Acsomycota, 400MYA, B = Bilateria 550MYA and C = Streptophytes 475 MYA. P = Paleozoic; M = Mesozoic; C = Cenozoic; Camb. = Cambrian. Click here for file

Additional file 4 **Partial GSII_B _sequences were identified in two additional Chloroplastida by preliminary phylogenetic analyses**. Partial GSII_B _sequences were obtained for *Acetabularia acetabulum *(Chlorophyta: Ulvophyceae) and *Mesostigma viride *(Streptophyta: Mesostigmatophyceae). These sequences were not included in the phylogenetic analyses presented within the paper (figures 1, 2, 3 and 4) due to their short length. Phylogenetic analysis confirming these proteins as members of the GSII_B _clade is presented in Additional file 2. Click here for file

Additional file 5 **Predicted cellular localization of GSII proteins in early-diverging Chloroplastida**. Only taxa containing both GSII_B _and GSII_E _within Chloroplastida were analyzed. N-terminal sequences of GSII proteins were analyzed for organellar transit peptides, *in silico*. References are given for established functional localizations. Click here for file
