# Supplementary material for: Molecular evolution of glutamine synthetase II: Phylogenetic evidence of a non-endosymbiotic gene transfer event early in plant evolution
Source: BMC Evol Biol. 2010 Jun 25;10:198. doi: 10.1186/1471-2148-10-198 (PMC2978018; doi:10.1186/1471-2148-10-198)
Supplement: Additional file 1 — GSII protein sequences used in the present study. GenBank accession numbers and JGI DOE scaffold and protein ID information for the GSII proteins are provided. [file 1471-2148-10-198-S1.DOC]

S. I. TABLE 1. Information for GSII protein sequences from organisms used in the present study. GenBank accession numbers and JGI DOE scaffold and protein ID information for the GSII proteins are provided.

|  | **GSII isoenzyme** | | |
| --- | --- | --- | --- |
| **Taxa** | **Accession Number/Protein ID** | | |
| **EUBACTERIA** |  |  |  |
| **Bacteroidetes** |  |  |  |
| *Cellulophaga* sp. MED134 | ZP_01050835.1 |  |  |
| *Flavobacterium* sp. MED217 | ZP_01059790.1 |  |  |
| *Polaribacter irgensii* 23-P | ZP_01119343.1 |  |  |
| *Robiginitalea biformata* HTCC2501 | ZP_01121593.1 |  |  |
| *Tenacibaculum* sp. MED152 | ZP_01052805.1 |  |  |
| *Flavobacteriales bacterium* ALC-1 | 641380439.NZ_ABHI01000002 (JGI-DOE-IMG) | ZP_02182161.1 | EDP71659.1 |
| *Kordia algicida* OT-1 | 641380434.NZ_ABIB01000013 (JGI-DOE-IMG) | ZP_02163676.1 | EDP94795.1 |
| *Psychroflexus torquis* ATCC 700755 | 638341165.NZ_AAPR01001082 (JGI-DOE-IMG) | NCBI/RefSeq: |  |
|  |  | NZ_AAPR01001082 |  |
| **Bacteroidetes** |  |  |  |
| **Flavobacteria** |  |  |  |
| *Flavobacterium johnsoniae* | ABQ05371.1 |  |  |
| **Bacteroidetes** |  |  |  |
| **Cytophaga** |  |  |  |
| *Cytophaga hutchinsonii* | ABG59001.1 |  |  |
| **Planctomycetes** |  |  |  |
| *Rhodopirellula baltica* | CAD75267.1 |  |  |
| *Fulvimarina pelagi* HTCC2506 | 639857001.NZ_AATP01000001(JGI-DOE-IMG) | ZP_01437475.1 | EAU42472.1 |
| **Verrucomicrobia** |  |  |  |
| *Opitutus terrae* | ACB77839.1 |  |  |
| *Chthoniobacter flavus* Ellin428 | 642791618.NZ_ABVL01000021(JGI-DOE-IMG) | ZP_03131969.1 | EDY17289.1 |
| *Opitutaceae bacterium* TAV2 | 640963002.NZ_ABEA01000149 (JGI-DOE-IMG) | ZP_03726965.1 | EEG19014.1 |
| *Verrucomicrobium spinosum* DSM 4136 | 641736179.NZ_ABIZ01000001(JGI-DOE-IMG) | ZP_02929155.1 |  |
| **Actinobacteria** |  |  |  |
| *Streptomyces coelicolor* | CAB43949.1 |  |  |
| *Streptomyces avermitilis* | BAC73709.1 |  |  |
| *Streptomyces viridochromogenes* | CAA37028.1 |  |  |
| *Streptomyces griseus* subsp. *griseus* | BAG22131.1 |  |  |
| *Streptomyces hygroscopicus* | AAA26749.1 |  |  |
| *Frankia alni* | AAA62803.1 |  |  |
| *Frankia alni* | CAJ63803.1 |  |  |
| *Frankia* sp. | ABD12500.1 |  |  |
| *Frankia* sp. | ABW11207.1 |  |  |
| *Frankia* sp. ACN14a | AAD53242.1 |  |  |
| **Proteobacteria** |  |  |  |
| **Alpha-proteobacteria** |  |  |  |
| *Methylobacterium nodulans* | ACL59375.1 |  |  |
| *Agrobacterium tumefaciens* | AAK88152.2 |  |  |
| *Ochrobactrum anthropi* | ABS16857.1 |  |  |
| *Methylobacterium populi* | ACB80494.1 |  |  |
| *Rhodopseudomonas palustris* | ABD06115.1 |  |  |
| *Rhizobium leguminosarum* | CAA47710.1 |  |  |
| *Bradyrhizobium* sp. | CAL77185.1 |  |  |
| *Sinorhizobium meliloti* | CAC49837.1 |  |  |
| *Rhodopseudomonas palustris* | ABD87101.1 |  |  |
| *Methylocella silvestris* | ACK49400.1 |  |  |
| *Rhizobium etli* | ACE92211.1 |  |  |
| *Xanthobacter autotrophicus* | ABS66390.1 |  |  |
| *Mesorhizobium loti* | BAB47942.1 |  |  |
| *Agrobacterium* sp. | ABU49125.1 |  |  |
| *Rhizobium tropici* | ABW74207.1 |  |  |
| *Bradyrhizobium japonicum* | BAC49434.1 |  |  |
| *Sinorhizobium saheli* | AAF20036.1 |  |  |
| *Sinorhizobium meliloti* | ABW74199.1 |  |  |
| *Mesorhizobium plurifarium* | ABU49127.1 |  |  |
| *Bradyrhizobium elkanii* | ABW74198.1 |  |  |
| *Mesorhizobium tarimense* | ABY73749.1 |  |  |
| **Proteobacteria** |  |  |  |
| **Gamma-proteobacteria** |  |  |  |
| *Coxiella burnetii* | ACJ20530.1 |  |  |
| *Francisella tularensis* subsp. *tularensis* | ABO47544.1 |  |  |
| *Francisella philomiragia* subsp. *philomiragia* | ABZ86871.1 |  |  |
| **EUKARYOTES** |  |  |  |
| **Opisthokonts** |  |  |  |
| **Fungi** |  |  |  |
| *Colletotrichum gloeosporioides* | AAB00322.1 |  |  |
| *Neurospora crassa* OR74A | EAA29877.2 |  |  |
| *Gibberella fujikuroi* | CAC27836.1 |  |  |
| *Schizosaccharomyces pombe* | CAB11660.1 |  |  |
| *Saccharomyces cerevisiae* | NP_015360.1 |  |  |
| *Kluyveromyces lactis* | CAD67983.1 |  |  |
| *Hebeloma cylindrosporum* | AAK96111.1 |  |  |
| *Agaricus bisporus* | CAA73235.1 |  |  |
| *Suillus bovinus* | CAD48934.1 |  |  |
| *Amanita muscaria* | CAD22045.1 |  |  |
| *Schizophyllum commune* | AAF27660.1 |  |  |
| *Cryptococcus neoformans* var. *neoformans* | CAD10037.1 |  |  |
| *Candida albicans* SC5314 | XP_711992.1 |  |  |
|  |  |  |  |
| **Metazoa** |  |  |  |
| *Danio rerio* | NP_878286.1 |  |  |
| *Oncorhynchus mykiss* | AAM73659.1 |  |  |
| *Mus musculus* | CAA34381.1 |  |  |
| *Opsanus beta* | AAN77155.1 |  |  |
| *Xenopus laevis* | DAA00256.1 |  |  |
| *Paracentrotus lividus* | AAC41562.1 |  |  |
| *Drosophila melanogaster* | AAN09632.1 |  |  |
| *Drosophila melanogaster* | AAF48043.2 |  |  |
| *Aedes aegypti* | AAK76447.1 |  |  |
| *Panulirus argus* | AAA02583.1 |  |  |
| *Schistosoma japonicum* | AAP06276.1 |  |  |
| *Anopheles gambiae* str. PEST | EAA08219.4 |  |  |
| *Caenorhabditis elegans* | CAA82655.1 |  |  |
|  |  |  |  |
| **Chromalveolates** |  |  |  |
| **Heterokonts(Stramenopiles)** |  |  |  |
| **Diatoms** |  |  |  |
| *Skeletonema costatum* | AAC77446.1 |  |  |
| *Phaeodactylum tricornutum* CCAP 1055/1 | XP_002182209.1 |  |  |
| *Thalassiosira pseudonana* JGI DOE | estExt_fgenesh1_kg.C_chr_220004 JGI DOE | 26051 |  |
| **Pelagophyte** |  |  |  |
| *Aureococcus anopagefferens* | e_gw1.2.832.1 JGI-DOE | 20700 |  |
| **Oomycetes** |  |  |  |
| *Phytophthora capsici* | estExt_fgenesh1_kg.C_130491 JGI-DOE | 100654 |  |
| *Phytophthora capsici* | estExt_Genewise1Plus.C_130172 JGI-DOE | 36513 |  |
| *Phytophthora ramorum* | gwEuk.94.20.1 JGI-DOE | 54729 |  |
| *Phytophthora infestans* | AAN31463.1 |  |  |
| *Leptolegnia chapmanii* | ABA60428.1 |  |  |
| *Lagenidium giganteum* | ABA60427.1 |  |  |
| *Aphanomyces euteiches* | CU357664.1 |  |  |
| **Haptophytes** |  |  |  |
| *Isochrysis galbana* | ABD94151.1 |  |  |
| *Emiliania huxleyi* CCMP1516 | e_gw1.97.36.1 JGI-DOE | 69253 |  |
| *Emiliania huxleyi* CCMP1516 | estExtDG_fgenesh_newKGs_kg.C_550058 JGI-DOE | 437187 |  |
|  |  |  |
| *Prymnesium parvum* | DV103374.1 |  |  |
|  |  |  |  |
| **Chlorarachniophytes** |  |  |  |
| *Bigelowiella natans* | DR041061.1 |  |  |
| *Bigelowiella natans* | DR039992.1 |  |  |
| **Rhodophytes** |  |  |  |
| *Gelidium crinale* | AAK60408.1 |  |  |
| *Cyanidioschyzon merolae* | AP006491.2 |  |  |
| *Galdieria sulphuraria* | contigs from BLAST from http://genomics.msu.edu/galdieria |  |  |
|  |  |  |
| **Glaucocystophyceae** |  |  |  |
| *Cyanophora paradoxa* | Contigs made by SGR from following NCBI EST data |  |  |
|  |  |  |
|  | EG943761.1, EG945765.1, ES229334.1, ES231243.1, ES234137.1, ES234137.1, ES234880.1, EC660939.1, EC658137.1, EC654969.1, EC659664.1, EC657152.1, EC654149.1, EC654260.1 |  |  |
| **Viridiplantae** |  |  |  |
| **Chlorophyta** |  |  |  |
| **Chlorophyceae** |  |  |  |
| *Chlamydomonas incerta* | Contigs made by SGR from following NCBI EST data |  |  |
|  |  |  |
|  | EC114820, EC113855, EC113408, EC114456, EC115875, EC115814, EC116471, EC113475, EC113170, EC115650, EC113622, EC115541, EC113253, EC115717, EC116183, EC113168, EC113122, EC112843 |  |  |
|  |  |  |
|  |  |  |
|  |  |  |
|  |  |  |
|  |  |  |
|  |  |  |  |
| *Chlamydomonas reinhardtii (1)* | Q42689.1 |  |  |
| *Chlamydomonas reinhardtii (2)* | estExt_gwp_1W.C_180099 JGI-DOE | 136895 |  |
| *Chlamydomonas reinhardtii (3)* | Q42688.1 |  |  |
| *Dunaliella salina* | X15280.1 |  |  |
| *Dunaliella tertiolecta* | AAC77379.1 |  |  |
| *Polytomella parva* | Contigs made by SGR from following NCBI EST data |  |  |
|  |  |  |
|  | EC750978, EC750480, EC751269, EC750918, EC751268, EC743206, EC743338, EC743307, EC750918, EC743223 |  |  |
|  |  |  |
|  |  |  |
| *Scenedesmus obliquus* | Contigs made by SGR from following NCBI EST data |  |  |
|  |  |  |
|  | EC188528, EC187923, EC189023, EC188518, EC189249 |  |  |
|  |  |  |
|  |  |  |  |
| *Volvox carteri* f. *nagariensis (1)* | estExt_Genewise1.C_60346 JGI-DOE | 73314 |  |
| *Volvox carteri* f. *nagariensis (2)* | estExt_Genewise1.C_580116 JGI-DOE | 77041 |  |
| *Volvox carteri* f. *nagariensis (3)* | estExt_fgenesh4_pg.C_60228 JGI-DOE | 103492 |  |
| **Prasinophyceae** |  |  |  |
| *Scherffelia dubia* SAG 40.89 | AJ919795 + AJ919858 |  |  |
| **Trebouxiophyceae** |  |  |  |
| *Chlorella* sp. NC64A (1) | IGS.gm_6_00005 JGI-DOE | 143431 |  |
| *Chlorella* sp. NC64A (2) | estExt_fgenesh3_pm.C_30035 JGI-DOE | 56005 |  |
| *Chlorella vulgaris* C-169 *(1)* | estExt_Genewise1.C_70343 JGI-DOE | 37265 |  |
| *Chlorella vulgaris* C-169 *(2)* | estExt_Genewise1.C_60393 JGI-DOE | 37014 |  |
| *Pseudochlorella* sp. CCAP211/1A (1) | GQ491030 |  |  |
| *Pseudochlorella* sp. CCAP211/1A (2) | GQ465769 |  |  |
| *Chlorella luteoviridis* | GQ465770 |  |  |
| *Auxenochlorella protothecoides* | GQ465771 |  |  |
| *Prototheca zopfii* | GQ465772 |  |  |
| *Helicosporidium* sp. ex *Simulium jonesii* | ABC54740.1 |  |  |
| **Streptophyta** |  |  |  |
| **Zygnemophyceae** |  |  |  |
| **Desmidiales** |  |  |  |
| 1 *Closterium peracerosum-* | Contigs made by SGR from following NCBI EST data |  |  |
| *strigosum-littorale* complex |  |  |
|  | BW647474.1, AU295784.1, AU295121.1, BW648094.1, AU295399.1, BW648051.1 |  |  |
|  |  |  |
| 2 *Closterium peracerosum-* | Contigs made by SGR from following NCBI EST data |  |  |
| *strigosum-littorale* complex |  |  |
|  | BW647499.1, BW646757.1 |  |  |
| **Streptophytina** |  |  |  |
| **Embyrophyta** |  |  |  |
| **Bryophyta** |  |  |  |
| *Physcomitrella patens* subsp *patens (1)* | estExt_fgenesh1_pg.C_190154 JGI DOE | 160180 |  |
| *Physcomitrella patens* subsp *patens* (2) | estExt_gwp_gw1.C_3450001 JGI DOE | 198924 |  |
| *Physcomitrella patens* subsp *patens* (3) | estExt_fgenesh1_pm.C_3450002 JGI DOE | 108913 |  |
| *Physcomitrella patens* subsp *patens* (4) | e_gw1.241.101.1 JGI DOE | 146278 |  |
| *Physcomitrella patens* subsp *patens* (5) | e_gw1.40.194.1 JGI DOE | 122526 |  |
| **Marchantiophyta** |  |  |  |
| 1 *Marchantia polymorpha* | Contigs made by SGR from following NCBI EST data |  |  |
|  |  |  |
|  | BJ854233.1, BJ851733.1, BJ845414.1, BJ855459.1, BJ843260.1, BJ851687.1, BJ856698.1, BJ841669.1, BJ849792.1, BJ854662.1, BJ864242.1, BJ864403.1, BJ855942.1, BJ848638.1 |  |  |
|  |  |  |
|  |  |  |
|  |  |  |
|  |  |  |
| 2 *Marchantia polymorpha* | Contigs made by SGR from following NCBI EST data |  |  |
|  |  |  |
|  | BJ855984.1, BJ866696.1, BJ858978.1, BJ871782.1, BJ865191.1, BJ862023.1, BJ847797.1, BJ851829.1, BJ859876.1, BJ868326.1, BJ862529.1, BJ860021.1, BJ872836.1 |  |  |
|  |  |  |
|  |  |  |
|  |  |  |
|  |  |  |
| 3 *Marchantia polymorpha* | Contigs made by SGR from following NCBI EST data |  |  |
|  |  |  |
|  | BJ844163.1, BJ852387.1, BJ849253.1, BJ867307.1 BJ842473.1, BJ859648.1, BJ852022.1, BJ847314.1, BJ847700.1, BJ841646.1, BJ842465.1,BJ845674.1, BJ845247.1, BJ849565.1, BJ857204.1, BJ853276.1, BJ856243.1, BJ849138.1, BJ857104.1, BJ845764.1 |  |  |
|  |  |  |
|  |  |  |
|  |  |  |
|  |  |  |
|  |  |  |
|  |  |  |
| **Tracheophyta** |  |  |  |
| **Euphyllophyta** |  |  |  |
| **Spermatophyta** |  |  |  |
| *Arabidopsis thaliana* | AAL91141.1 |  |  |
|  | AAM91149.1 |  |  |
|  | NP_198576.1 |  |  |
|  |  |  |  |
| *Brassica napus* | CAA51280.1 |  |  |
|  | CAA73064.1 |  |  |
|  | CAA73063.1 |  |  |
|  | CAA73062.1 |  |  |
|  | CAA58118.1 |  |  |
|  | CAA54151.1 |  |  |
|  |  |  |  |
| *Glycine max* | AAX18865.1 |  |  |
|  | AAX18864.1 |  |  |
|  | AAK43833.1 |  |  |
|  | AAG24873.1 |  |  |
|  | AAC97935.1 |  |  |
|  | CAA57216.1 |  |  |
|  | CAA57346.1 |  |  |
|  | AAB23379.1 |  |  |
|  |  |  |  |
| *Populus trichocarpa* | ABK95973.1 |  |  |
|  | ABK95264.1 |  |  |
|  | ABK94955.1 |  |  |
|  | ABK94916.1 |  |  |
|  | ABK93921.1 |  |  |
|  | ABK93563.1 |  |  |
|  |  |  |  |
| *Pisum sativum* | CAJ87510.1 |  |  |
|  | CAA29057.1 |  |  |
|  | AAA33669.1 |  |  |
|  | AAA33653.1 |  |  |
|  | CAA28456.1 |  |  |
|  |  |  |  |
| *Oryza sativa* Japonica Group | BAH01417.1 |  |  |
|  | BAG97064.1 |  |  |
|  | BAG94045.1 |  |  |
|  | NP_001054133.1 |  |  |
|  |  |  |  |
| *Triticum aestivum* | AAZ80474.1 |  |  |
|  | AAZ30061.1 |  |  |
|  | AAZ30060.1 |  |  |
|  | AAZ30059.1 |  |  |
|  | AAZ30058.1 |  |  |
|  | AAZ30057.1 |  |  |
|  | AAR84350.1 |  |  |
|  | AAR84349.1 |  |  |
|  | AAR84348.1 |  |  |
|  | AAR84347.1 |  |  |
|  |  |  |  |
| *Zea mays* | ACG47508.1 |  |  |
|  | ACG35376.1 |  |  |
|  | ACG34436.1 |  |  |
|  | ACF84604.1 |  |  |
|  | ACF83467.1 |  |  |
|  | BAA03432.1 |  |  |
|  | CAA46724.1 |  |  |
|  | CAA46720.1 |  |  |
|  | CAA46719.1 |  |  |
|  | BAA03431.1 |  |  |
|  | BAA03430.1 |  |  |
|  |  |  |  |
| *Saccharum officinarum* | AAW21275.1 |  |  |
|  | AAW21274.1 |  |  |
|  | AAW21273.1 |  |  |
|  |  |  |  |
| *Phragmites australis* | BAD12059.1 |  |  |
|  | BAD12057.1 |  |  |
|  |  |  |  |
| *Picea sitchensis* | ABR17424.1 |  |  |
|  | ABK23372.1 |  |  |
|  |  |  |  |
| *Pinus sylvestris* | CAA06383.1 |  |  |
|  | CAA49476.1 |  |  |
|  | CAA52448.1 |  |  |
|  |  |  |  |
| **Lycopodiophyta** |  |  |  |
| *Selaginella moellendorffii* | estExt_Genewise1.C_340051 JGI-DOE | 151817 |  |
|  | estExt_Genewise1Plus.C_980204 JGI-DOE | 184683 |  |
|  | e_gw1.4.324.1 JGI-DOE | 82032 |  |
|  | estExt_fgenesh1_kg.C_180021 JGI-DOE | 270944 |  |
|  |  |  |  |
