# Supplementary figures and images for: Molecular evolution of glutamine synthetase II: Phylogenetic evidence of a non-endosymbiotic gene transfer event early in plant evolution
Source: BMC Evol Biol. 2010 Jun 25;10:198. doi: 10.1186/1471-2148-10-198 (PMC2978018; doi:10.1186/1471-2148-10-198)

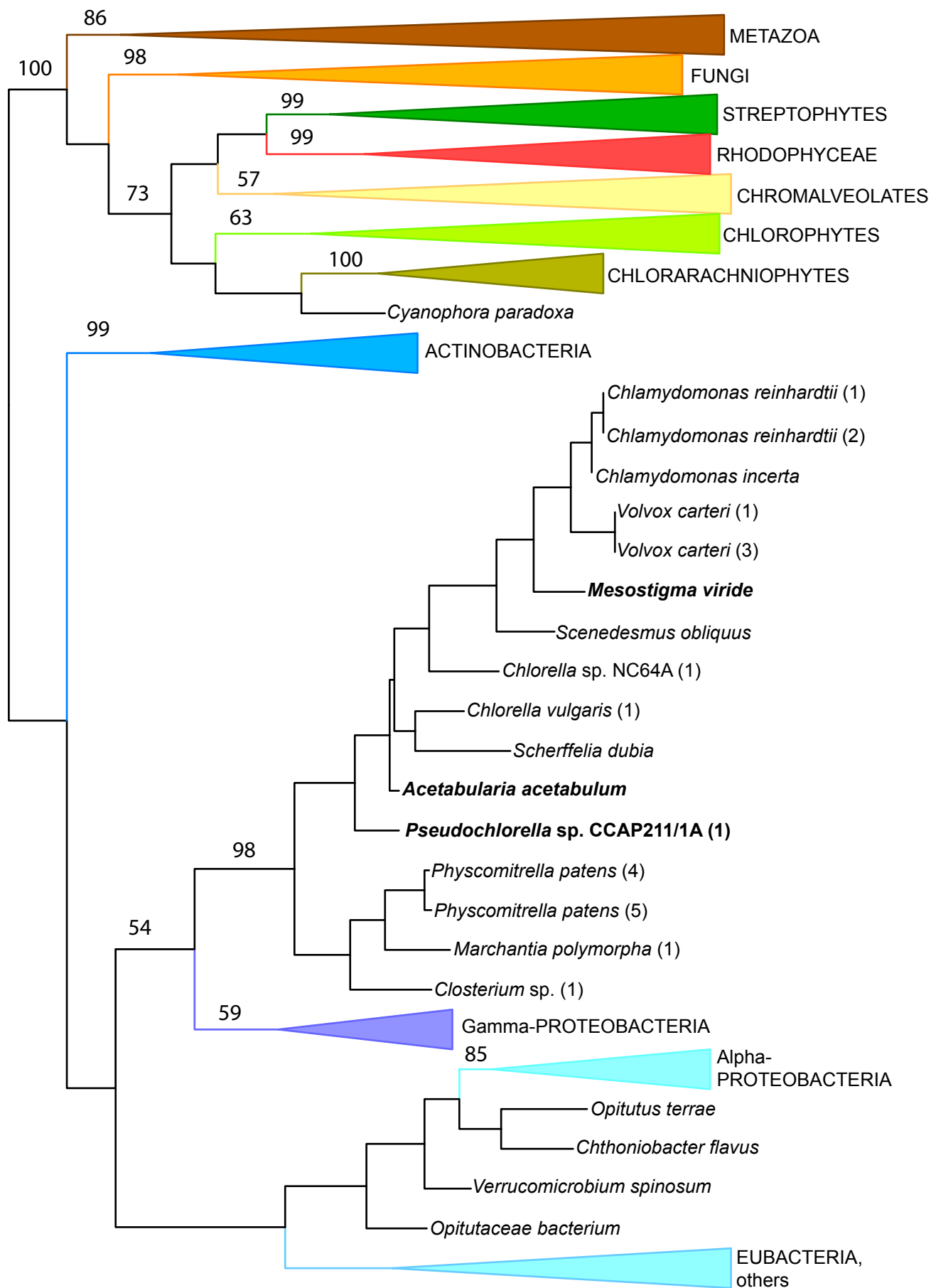

Supplement: Additional file 2 — Identification of GSIIB sequences from Acetabularia acetabulum and Mesostigma viride. The data matrix used in this analyses is described in the Methods with sequences from the following taxa included in the alignment: Acetabularia acetabulum (Chlorophyta, Ulvophyceae), Mesostigma viride (Streptophyta, Mesostigmatophyceae), which are shown in bold. Pseudochlorella sp. CCAP211/1A (1) GSIIB amplified in the present study is also shown in bold. Tree used for this illustration is a representative derived from parsimony heuristic search analysis. RAxML bootstrap values are shown for the major clades of GSII genes, which were derived from 1000 bootstrap replicates with the parameters described in the Methods. [file 1471-2148-10-198-S2.PDF]

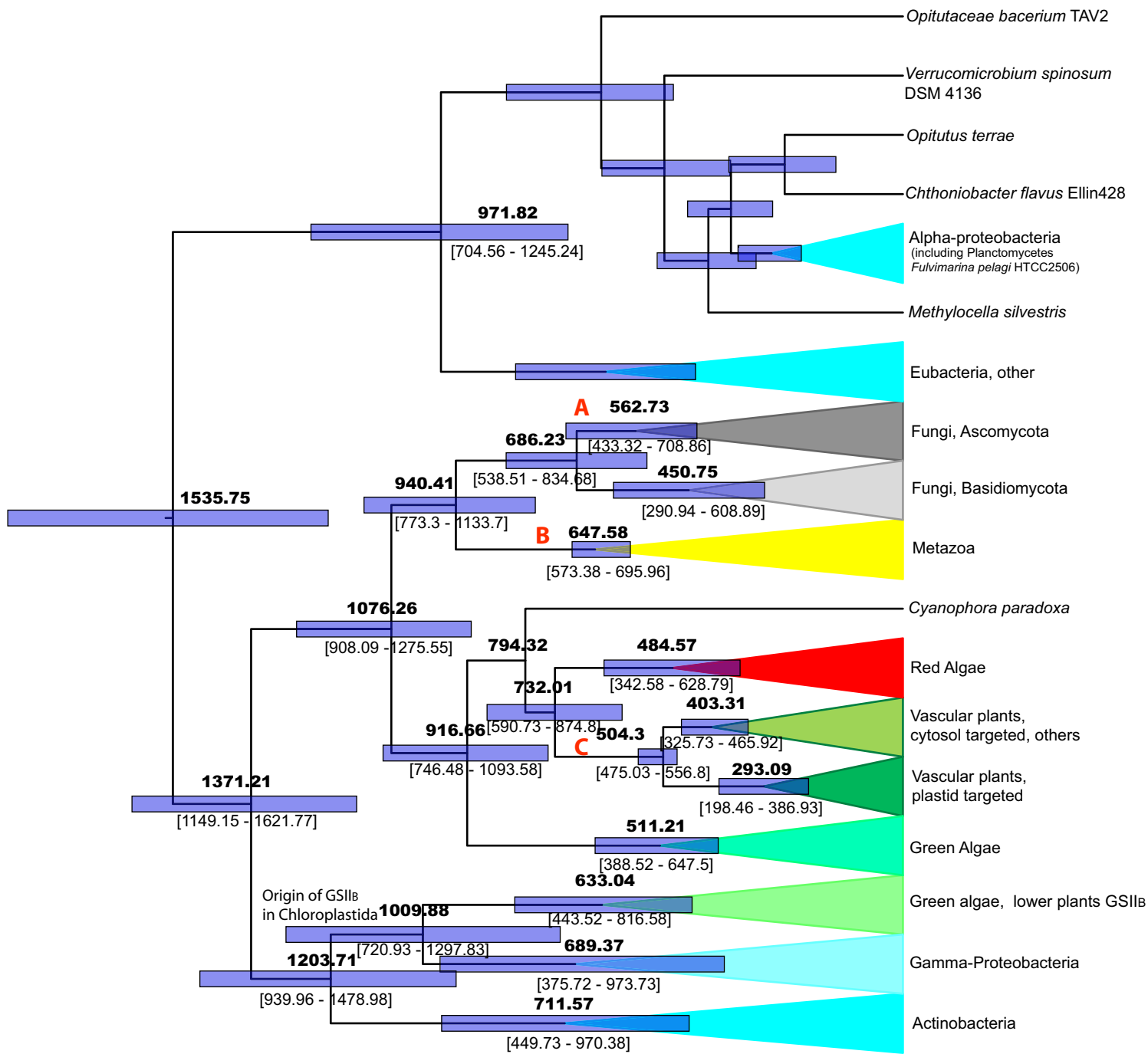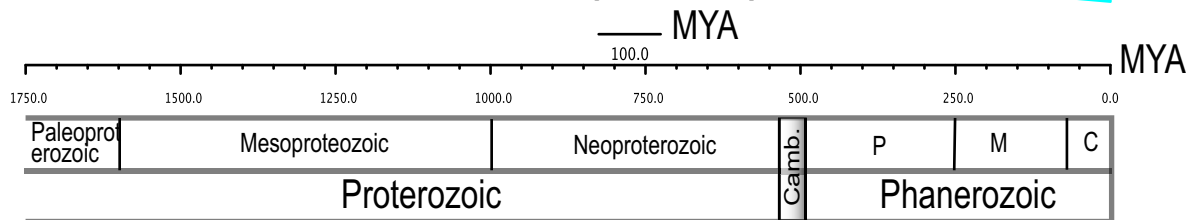

Supplement: Additional file 3 — Maximum clade probability tree displayed as a chronogram from the BEAST analysis of the GS amino acid sequence alignment. All lineages evolved according to a relaxed molecular clock and WAG + Inv. + Gamma model. Node bars indicate the width of the 95% highest posterior density with minimum and maximum values in parentheses. Bold numbers near the nodes indicate node ages. Major lineages are depicted as collapsed triangles. Nodes for which fossil dates were used are marked as A = Acsomycota, 400MYA, B = Bilateria 550MYA and C = Streptophytes 475 MYA. P = Paleozoic; M = Mesozoic; C = Cenozoic; Camb. = Cambrian. [file 1471-2148-10-198-S3.PDF]
