# Supplementary material for: Molecular evolution of glutamine synthetase II: Phylogenetic evidence of a non-endosymbiotic gene transfer event early in plant evolution
Source: BMC Evol Biol. 2010 Jun 25;10:198. doi: 10.1186/1471-2148-10-198 (PMC2978018; doi:10.1186/1471-2148-10-198)
Supplement: Additional file 4 — Partial GSIIB sequences were identified in two additional Chloroplastida by preliminary phylogenetic analyses. Partial GSIIB sequences were obtained for Acetabularia acetabulum (Chlorophyta: Ulvophyceae) and Mesostigma viride (Streptophyta: Mesostigmatophyceae). These sequences were not included in the phylogenetic analyses presented within the paper (figures 1, 2, 3 and 4) due to their short length. Phylogenetic analysis confirming these proteins as members of the GSIIB clade is presented in Additional file 2. [file 1471-2148-10-198-S4.DOC]

S. I. TABLE 2. Partial GSII_B_ sequences were identified in two additional Chloroplastida by preliminary phylogenetic analyses. Partial GSII_B_ sequences were obtained for *Acetabularia* *acetabulum* (Chlorophyta: Ulvophyceae) and *Mesostigma* *viride* (Streptophyta: Mesostigmatophyceae). These sequences were not included in the phylogenetic analyses presented within the paper (figures 1-4) due to their short length. Phylogenetic analysis confirming these proteins as members of the GSII_B_ clade are presented in Additional figure 5.
.

| **Taxa** | **GenBank** | **Taxonomy** |
| --- | --- | --- |
|  | **Accession number** |  |
|  |  |  |
| ***Acetabularia acetabulum*** | EC095027.1 | Viridiplantae; Chlorophyta; Ulvophyceae |
|  | EC095930.1 |  |
|  |  |  |
|  |  |  |
| ***Mesostigma viride*** | EC728058.1 | Viridiplantae; Streptophyta; |
|  | EC727990.1 | Mesostigmatophyceae |
