# Supplementary material for: Molecular evolution of glutamine synthetase II: Phylogenetic evidence of a non-endosymbiotic gene transfer event early in plant evolution
Source: BMC Evol Biol. 2010 Jun 25;10:198. doi: 10.1186/1471-2148-10-198 (PMC2978018; doi:10.1186/1471-2148-10-198)
Supplement: Additional file 5 — Predicted cellular localization of GSII proteins in early-diverging Chloroplastida. Only taxa containing both GSIIB and GSIIE within Chloroplastida were analyzed. N-terminal sequences of GSII proteins were analyzed for organellar transit peptides, in silico. References are given for established functional localizations. [file 1471-2148-10-198-S5.DOC]

S. I. TABLE 3. Identification of functional localizations of GSII proteins in basal Chloroplastida. Only taxa containing both GSII_B_ and GSII_E_ within Chloroplastida were analyzed. N-terminal sequences of GSII proteins were analyzed for organellar transit peptides, in silico. References are given for established functional localizations.

Table 3a. Information for GSII_B_ protein in basal Chloroplastida.

| **TAXA** | **GSIIB** | | | |
| --- | --- | --- | --- | --- |
|  | **Accession Number/** |  | **Functional localization** | **References** |
|  | **Protein ID** | |  |  |
| ***Chlamydomanas incerta*** | NCBI EST contig (SI Table 2) |  | - | Partial sequence, unable to predict |
|  |  |  |  |  |
| ***Chlamydomonas reinhardtii*** | Q42689.1 |  | Chloroplast/Mitochondria | Chen, Q. and Silflow, C.D.(1996)/ TargetP 1.1 |
|  |  |  |  |  |
|  | estExt_gwp_1W.C_180099 JGI-DOE | 136895 | Chloroplast/Mitochondria | Plastid precursor; JGI DOE/ TargetP 1.1 |
|  |  |  |  |  |
| ***Scenedesmus obliquus*** | NCBI EST contig (SI Table 2) |  | Mitochondria | TargetP 1.1 |
|  |  |  |  |  |
| ***Volvox carteri* f. *nagariensis*** | estExt_Genewise1.C_60346 JGI-DOE | 73314 | Mitochondria | TargetP 1.1 |
|  | estExt_fgenesh4_pg.C_60228 JGI-DOE | 103492 | Mitochondria | TargetP 1.1 |
|  |  |  |  |  |
| ***Scherffelia dubia* SAG 40.89** | AJ919795 + AJ919858 |  | - | Partial sequence, unable to predict |
|  |  |  |  |  |
| ***Chlorella* sp. NC64A** | IGS.gm_6_00005 JGI-DOE | 143431 | Chloroplast | ChloroP 1.1 |
|  |  |  |  |  |
| ***Chlorella vulgaris* C-169** | estExt_Genewise1.C_70343 JGI-DOE | 37265 | Chloroplast | ChloroP 1.1 |
|  |  |  |  |  |
| ***Pseudochlorella* sp. CCAP211/1A** | GQ491030 |  | - | Partial sequence, unable to predict |
|  |  |  |  |  |
| ***Closterium peracerosum-strigosum-littorale* complex (1)** | NCBI EST contig (SI Table 2) |  | Chloroplast | ChloroP 1.1 |
|  |  |  |  |
|  |  |  |  |  |
| ***Physcomitrella patens* subsp *patens*** | e_gw1.241.101.1 JGI DOE | 146278 | Cytosol^±^ | TargetP 1.1 |
|  | e_gw1.40.194.1 JGI DOE | 122526 | Cytosol^±^ | TargetP 1.1 |
|  |  |  |  |  |
|  |  |  |  |  |
| ***Marchantia polymorpha* (1)** | NCBI EST contig (SI Table 2) |  | - | Partial sequence, unable to predict |
|  |  |  |  |  |

Table 3b. Information for GSII_E_ protein in basal Chloroplastida.

| **TAXA** | **GSIIE** | | | |
| --- | --- | --- | --- | --- |
|  | **Accession Number/Protein ID** | | **Functional localization** | **References** |
| ***Chlamydomonas incerta*** | N. D. |  | - | - |
|  |  |  |  |  |
| ***Chlamydomonas reinhardtii*** | Q42688.1 |  | Cytosol^±^ | Chen, Q. and Silflow, C.D.(1996)/ TargetP 1.1 |
|  |  |  |  |  |
| ***Scenedesmus obliquus*** | N. D. |  | - | - |
|  |  |  |  |  |
| ***Volvox carteri* f*. nagariensis*** | estExt_Genewise1.C_580116 JGI-DOE | 77041 | Cytosol^±^ | TargetP 1.1 |
|  |  |  |  |  |
| ***Scherffelia dubia* SAG 40.89** | N. D. |  | - | - |
|  |  |  |  |  |
| ***Chlorella* sp. NC64A** | estExt_fgenesh3_pm.C_30035 JGI-DOE | 56005 | Cytosol^±^ | TargetP 1.1 |
|  |  |  |  |  |
| ***Chlorella vulgaris* C-169** | estExt_Genewise1.C_60393 JGI-DOE | 37014 | Cytosol^±^ | TargetP 1.1 |
|  |  |  |  |  |
| ***Pseudochlorella* sp. CCAP211/1A** | GQ465769 |  | Cytosol^±^ | TargetP 1.1 |
|  |  |  |  |  |
| ***Closterium peracerosum-strigosum-littorale* complex (2)** | NCBI EST contig (SI Table 2) |  | Cytosol^±^ | TargetP 1.1 |
|  |  |  |  |
|  |  |  |  |  |
| ***Physcomitrella patens* subsp *patens*** | estExt_fgenesh1_pg.C_190154 JGI DOE | 160180 | Cytosol^±^ | TargetP 1.1 |
|  | estExt_gwp_gw1.C_3450001 JGI DOE | 198924 | Cytosol^±^ | TargetP 1.1 |
|  | estExt_fgenesh1_pm.C_3450002 JGI DOE | 108913 | Cytosol^±^ | TargetP 1.1 |
|  |  |  |  |  |
| ***Marchantia polymorpha* (2)** | NCBI EST contig (SI Table 2) |  | Cytosol^±^ | TargetP 1.1 |
| ***Marchantia polymorpha* (3)** | NCBI EST contig (SI Table 2) |  | Cytosol^±^ | TargetP 1.1 |

N. D. No Data

± In the absence of any evidence from N-terminal presequence as determined by TargetP and ChloroP these proteins are putatively assigned cytosolic function. The functional localization merits further investigation beyond the scope of the present research.
